# Evaluation of person-centred care within the Stepstones transition program for adolescents with congenital heart disease – a document analysis

**DOI:** 10.1016/j.ijnsa.2025.100308

**Published:** 2025-02-10

**Authors:** Åsa Burström, Markus Saarijärvi, Sandra Skogby, Anna Lena Brorsson, Ewa-Lena Bratt, Carina Sparud-Lundin

**Affiliations:** a Dept. Neurobiology, Care Science and Society, Karolinska Institutet, Stockholm, Sweden; b Department of Cardiology, Danderyd Hospital Corporation, Danderyd, Sweden; c Region Västra Götaland, Sahlgrenska University Hospital, Children's Heart Center, Gothenburg, Sweden; d Astrid Lindgren Children's Hospital, Karolinska University Hospital, Stockholm, Sweden; e Institute of Health and Care Sciences, University of Gothenburg, Gothenburg, Sweden

**Keywords:** Adolescents, Adolescent health, Chronic disease, Document analysis, Heart defects, Congenital, Patient-Centered care, Person-Centred care, Transfer of care, Transition of care

## Abstract

**Background:**

Transition programs are pivotal in ensuring successful transition to adulthood and transfer to adult care for adolescents with chronic conditions. Healthcare providers must therefore support adolescents in increasing their empowerment to gain active participation in health and care. The Stepstones transition program is based on a person-centred care approach and has been evaluated in a randomized controlled trial. However, the extent to which the person-centred care approach was implemented needs further exploration.

**Objective:**

To evaluate how a person-centred care approach was implemented based on a documentation analysis of the Stepstones transition program for adolescents with congenital heart disease.

**Design:**

A deductive qualitative design employing analysis of the documentation used in the Stepstones transition program.

**Setting(s):**

The documentation derives from the consultations in the two interventions centres in the Stepstones randomized controlled trial. The trial was conducted between 2017 and 2021. Data sources included in this study were: The adolescents’ written narratives, documentation of the person-centred conversations, and goalsetting in the transition plans.

**Participants:**

Documentation for adolescents with congenital heart disease, randomized to the intervention group (*n* = 59) at the two intervention centres was included.

**Methods:**

Directed content analysis was used to evaluate how person-centred care was described in the documentation. The analysis was based on the three cornerstones of person-centred care: initiating, establishing and safeguarding partnership, in this study between the adolescent and the transition coordinator during the transition program. The data were deductively sorted into aspects of relevance for person-centred care and adolescent health. For the written narratives an inductive analysis was thereafter undertaken.

**Results:**

A partnership between the transition coordinator and the adolescent was initiated through the adolescents’ written narrative and established using a psychosocial interview guide. This outlined a spectrum of aspects important to person-centred and adolescent-oriented approach, such as resources, risks/obstacles, and needs. The goalsetting process describes goals commonly agreed upon and how to accomplish them. A solid foundation of self-awareness regarding personal capacities and learning needs contributed to the development of knowledge, understanding, and fostered independence to various degrees.

**Conclusions:**

The Stepstones transition program for adolescents with congenital heart disease implemented several person-centred components, such as eliciting narratives, collaborative goal setting, and tailoring support needs. The documentation had limitations in fully reflecting the person-centred practices employed, highlighting opportunities for improvement in person-centred documentation.

**Tweetable abstract:**

“The study delves into the implementation of person-centred care in the Stepstones transition program for adolescents with congenital heart disease, revealing strengths and areas for improvement in documentation.”

**What is already known**

• Transition programs are important in supporting adolescents with chronic conditions to assume responsibility for their health, develop self-management skills, and gain control over their health.
• In person-centred care, the perspective changes from seeing the patient as a care recipient to seeing them as an active stakeholder in the care and decision-making process.
• The implementation of person-centred care within complex healthcare interventions is not a straightforward process and requires examination of intervention processes.

Alt-text: Unlabelled box

**What this paper adds**

• To achieve person-centred care for adolescents with chronic conditions in transition to adulthood, adolescent-oriented communication styles are pivotal.
• Person-centred care can help in finding goals to establish independence and self-management for adolescents in transition to adulthood.
• Documentary analysis can provide both depth and breadth in how person-centred care is implemented within complex healthcare interventions but should be complemented with other empirical data sources.

Alt-text: Unlabelled box

## 1 Background

International guidelines emphasize the significance of transition planning and transition programs for adolescents with chronic conditions (CC) as they transition to adulthood and transfer to adult care (Mazur et al., 2017; Medicine, 2020). The objective of transition programs is to prepare and empower adolescents with chronic conditions to assume responsibility for their health, develop self-management skills, and gain control over their illness (Cooley et al., 2011). Before adolescents with chronic conditions enter adulthood, they must begin to take charge of their health and well-being, preparing for the transfer to an adult healthcare setting (Medicine, 2020). As they gain autonomy and independence, they need to acquire the necessary knowledge and skills to actively manage their health and care, both in the present and in the future (Mazur et al., 2017). This involves not only taking control of their health but also mastering the complexity of the healthcare organization that this subsequent shift in care provider entails (Coyne et al., 2019; Kokorelias et al., 2023). Adolescence also marks a shift in the role of parents as they transition from primary caregivers to supportive and coaching people on their child's healthcare journey (Coyne et al., 2019; Heath et al., 2017). This critical period in an adolescent's life not only shapes their future health outcomes but also redefines the dynamics of family involvement.

To ensure a successful transition into adulthood and transfer to adult care, healthcare providers must educate and support adolescents in self-management to gain active participation in their care. Moreover, it is recommended that the transition process is individualized, with adolescents and parents as active partners, emphasizing shared decision-making in healthcare consultations (Mazur et al., 2017). Adolescents who experience good communication and a respectful, mutual relationship with healthcare providers are more likely to enhance their autonomy in managing their care (Johnson et al., 2015). Conversely, unprepared adolescents often report anxiety and feelings of inadequacy in participating in their care due to a lack of knowledge (Varty et al., 2020). However, there is considerable variation in experiences related to transition preparation, including the content and timing of the transfer for adolescents with chronic conditions (Coyne et al., 2019).

Transition programs have beneficial effects on disease-related self-management and knowledge, reducing non-adherence and increasing self-efficacy in adolescents with chronic conditions (Ravens et al., 2020). For adolescents with congenital heart disease (CHD), these programs also improve overall health perception (Flocco et al., 2019), transition readiness, empowerment (Moons et al., 2021), self-efficacy, and quality of life (John et al., 2022). Transition programs offer various components, such as nurse-led transition clinics (Berg and Hertz, 2007), nurse-led educational sessions (Mackie et al., 2018), meetings with transition coordinators (Flocco et al., 2019), tailored education, and support through discussions with peer counsellors and psychologists, as well as increased engagement of adolescents and their families, coordinated by transition coordinators (Flocco et al., 2019).

The Stepstones transition program is based on person-centred care and was evaluated through a randomized controlled trial (RCT) in adolescents with CHD. The primary outcome of the RCT was to investigate the empowering effect of a structured transition program with a person-centred care (PCC) approach. The findings revealed that the transition program effectively increased patient empowerment, transition readiness, disease-related knowledge, reduced parental involvement, and improved satisfaction with physical appearance (Bratt et al., 2023). The Stepstones transition program incorporated the three cornerstones of PCC based on the Gothenburg Centre for Person-Centred Care model (Ekman et al., 2011). These cornerstones include 1) initiating a partnership with the adolescent, 2) establishing the partnership through shared decision-making, and 3) safeguarding the partnership via joint documentation (Britten et al., 2017; Ekman et al., 2011).

Person-centred care is a concept that is of growing interest in healthcare, shifting the view of patients as passive recipients of care to one where they are seen as active participants who are invited to take an active role in their care and decision-making (Ekman et al., 2011). In PCC, the patient's narrative about how the disease or condition impacts their daily life is crucial, as is identifying their capabilities and resources (1). By engaging in discussions and sharing experiences, a partnership is established to build a common understanding for planning care and treatment and to achieve mutually agreed-upon goals (2). These need to be documented to safeguard the established partnership (3) (Ekman et al., 2011). The Stepstones program aspired to implement a PCC approach but the extent to which this approach was realized needs to be further explored.

The objective of this study therefore is to evaluate how a PCC approach was implemented based on a documentation analysis of the Stepstones transition program for adolescents with CHD. The guiding research questions were:

1) How could the adolescents’ narrative be captured through the personal story?
2) How was the partnership initiated, established and safeguarded, according to available documentation?

## 2 Methods

### 2.1 Design

A deductive qualitative design was employed with documentation analysis of three written pieces of documentation used in the Stepstones transition program and evaluated in an RCT (Bratt et al., 2023). Deductive qualitative analysis combines deductive and inductive analysis to examine supporting, contradicting, refining, and expanding evidence for the theory or conceptual model, (Fife, 2024) in this study PCC. The report followed the standards for reporting qualitative research (SRQR) (O'Brien et al., 2014).

#### 2.1.1 The study setting

The documentation derives from the consultations in the two interventions centres. Totally, seven paediatric cardiology centres (five control centres) in Sweden were included in the Stepstones RCT study. The Stepstones transition program was developed by a research group at University of Gothenburg in Sweden targeting adolescents with chronic conditions, adapted from a Belgian study for adolescents with rheumatic diseases (Hilderson et al., 2016). The development and adaptation of the transition program followed the Medical Research Council framework for complex interventions (Craig et al., 2008), as well as the protocol of intervention mapping, which involved stakeholder involvement, a needs assessment through preparatory studies, and a literature review (Acuña Mora et al., 2020). The transition program for adolescents with CHD was evaluated through a hybrid RCT in which an RCT was embedded within a longitudinal observational study. The evaluation of the program comprised an efficacy evaluation, health economic evaluation, and process evaluation (Acuna Mora et al., 2017; Saarijärvi et al., 2019). The efficacy evaluation of the RCT showed that the intervention (i.e. transition program in addition to usual care) was superior to controls (i.e. only usual care) in achieving the primary outcome, as well as several secondary outcome measures (Bratt et al., 2023). The process evaluation concluded that the intervention was implemented with high fidelity and patient satisfaction, and participants especially valued the meetings with the transition coordinator (TC) (Saarijärvi et al., 2022; Saarijärvi et al., 2021).

**Fig. 1:**
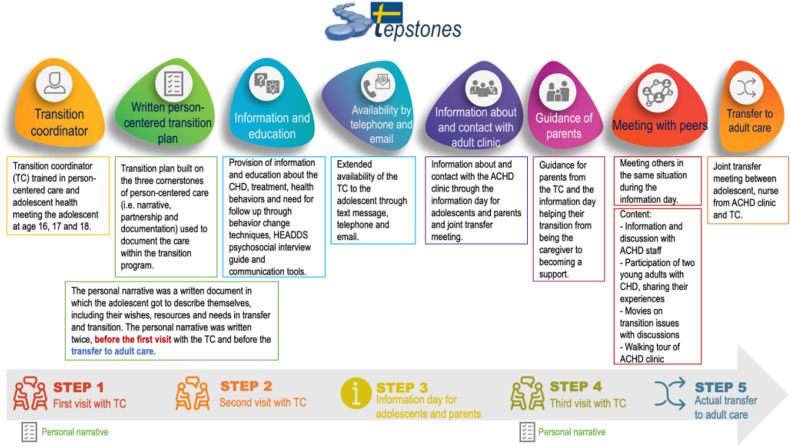
Components and implementation steps of the STEPSTONES transition program. HEADDS (Home Education Activities Depression Drugs Sexuality). TC=Transition coordinator. CHD = Congenital Heart Disease. ACHD= Adults with Congenital Heart Disease.

Source: reproduced with permission from BioMed Central Ltd. (Saarijärvi et al., 2022).

The transition program comprised eight components delivered in five steps (Fig. 1). The transition program was performed mainly by the TC, a specially trained clinical nurse specialist in adolescent health and trained in PCC. The TC met the adolescents in three consultations at ages 16, 17, and 18. Person-centred care is vital to endorse empowerment in adolescents to be active partners in their health and care and decision making.

In accordance with a PCC approach, the adolescents and TC developed a transition care plan. Before the first meeting (age 16) and before the last meeting (age 18) the adolescents were asked to write a personal narrative in which they described themselves, their view and resources, wishes and need in relation to transition and transfer to adult care.

The partnership between the adolescents and TC was initiated and developed during the conversations in the meetings. To facilitate conversation, the TCs were trained to use HEADDS (Home Education Activities Depression Diet Sexuality), a psychosocial interview guide developed within adolescent health and medicine to optimize communication about the context of adolescent life, identify risks and resources, and minimize stress and stigma (Cohen et al., 1991). A cornerstone of the interview conversation was to safeguard confidentiality between adolescent and healthcare provider. These conversations formed the starting point for a common understanding of the adolescent's health behaviour (including potential risk behaviour), the impact on the disease in daily life, as well as identification of their own resources and need for support. This was documented in the transition plan and followed up during next visit. The TCs used different tools to facilitate communication like concept maps and pictures etc. In the last step, the goals and care arrangements jointly agreed upon were documented in the transition plan and followed up during the following consultation (Supplementary file 1 – Transition plan).

### 2.2 Participants and recruitment

The sample in this study consisted of transition plans for adolescents participating in the intervention group of the RCT (*n* = 59). The inclusion criteria were adolescents with a CHD, 16 years of age at time for inclusion, Swedish speaking and literate. The exclusion criteria were cognitive or physical difficulties to complete the questionnaires of the outcome evaluation (not included in this study). A full description of the sampling procedure, as well as an analysis of recruitment, reach and retention are described in separate publications (Bratt et al., 2023; Saarijärvi et al., 2020).

### 2.3 Data collection

The RCT was conducted between 2017 and 2021 and the three data sources included in this study were collected as a part of the process evaluation.

The three data sources included were:

• Personal story (the adolescent-written narrative)
• Documentation of the person-centred conversation based on the use of HEADSS (a summary in the transition plan made by the TC)
• Documentation of jointly formulated goal setting (the adolescents together with the TC)

The TCs collecting the data in the RCT (Bratt et al., 2023) were two co-authors (SS and ÅB). The data were collected either in paper or electronically, transcribed and imported into a Microsoft Access Database by the researcher responsible for the process evaluation of the RCT (MS). In total, 89.8 % of the participants attended all three consultations with the TC. A more detailed description of the process evaluation variables related to PCC are shown in Table 1 and can be retrieved from a previous publication (Saarijärvi et al., 2022).

**Table 1:** Variables on the implementation fidelity of the Stepstones transition program.

Process evaluation variables of the PCC aspects*	Proportion (%) for each of the three consultations		
*Aspects of PCC*	Visit 1	Visit 2	*Visit 3*
Proportion of participants that wrote a personal narrative before the consultations ⁎⁎	89.8	N.A	55.9
Participants that had a goal set after the consultation	96.6	88.1	37.3
Resources identified in adolescents in relation to goalsetting	57.6	55.9	13.6
Identification of supportive networks performed	94.9	76.3	52.5
Willingness and need to participate in care addressed	96.6	93.2	89.8
Usage of HEADDS during the consultations	100	93.2	89.8
Personal narrative sent to adult care prior to last visit	-	-	5.1

⁎ Process evaluation variables in its’ entirety can be obtained in the reference by Saarijärvi, et al. (2022).

⁎⁎ according to the program and following the steps in PCC participants were advised to write this before the first and the third visit to the TC.

### 2.4 Data analysis

The analytic procedure in the documentation analysis entailed selecting, making sense of and synthesizing data contained in the three sources of documentation. A directed content analysis was used to assess the extent to which PCC was evident in the documentation (Hsieh and Shannon, 2005). The GPCC model guided the data analysis in the deductive approach, while newly identified categories were identified inductively to further refine, extend, and enrich the model (Fife, 2024). The analysis of the first and second source were built on key aspects of PCC and an adolescent-oriented care approach. These aspects were resources and capacities, needs and need of support (PCC), risks and obstacles (adolescent-oriented care). 1) The adolescents’ written narratives were initially transcribed into an excel sheet, deductively sorted into the aspects (SS and ÅB). An inductive analysis was then undertaken (SS and ÅB) whereby categories emerged and were negotiated among the co-authors (ALB, CSL, ELB and MS). 2) The analysis of the documentation in the transition plans, based on the conversation guided by the psychosocial interview guide HEADDS and its domains (Cohen et al., 1991), was inserted into the Nvivo software for qualitative data analysis (ALB and MS). 3) The analysis of the personal goals agreed upon in the transition plan were also conducted in Nvivo and sorted into the five dimensions of empowerment, knowledge and understanding, personal control, shared decision-making, identity, and enabling others (Small et al., 2013). Different aspects of these dimensions are depicted in the subcategories, followed by descriptions of how to accomplish these goals (CSL and ELB). Finally, the co-authors jointly discussed the procedures and content of analysis for all three sources of documentation, and their relation to the categories (based on the three cornerstones of PCC). Preunderstanding and its potential impact on the analysis was nevertheless considered, and because the two co-authors (SS and ÅB) had compiled the documentation in the transition plans they were not involved in the analyses of these sources. The authors discussed and scrutinized their pre-understanding throughout the analysis. Credibility was enhanced by applying this reflexive approach and by employing multiple data sources (triangulation).

### 2.5 Ethical considerations

The study was performed according to national ethical regulations (EPM, 2024) and the Declaration of Helsinki (World Medical, 2013). Our study participants agreed to participate after receiving verbal and written information about the study and signing a written consent form. To safeguard confidentiality all data were digitally stored in a password-protected computer at a safe server in the university database. Ethical approval was received from the Regional Ethical Review Board in Gothenburg, Sweden (No. 931–15).

## 3 Results

The analysis was based on the documentation generated within the Stepstones transition program, which included 59 adolescents (44.1 % female) with a variation of CHD severity (categorized according to Baumgartner et al., 2021); mild 15.3 %, moderate 52.5 %, and severe 32.2 %. Of these participants, 30,5 % had additional comorbidities.

The results are presented in relation to the three documentation sources, depicted in the three categories reflecting the cornerstones of PCC, (1), initiating establishing (2) and safeguarding (3) the partnership which is the core of PCC. An overview of the results, the categories, explored aspects, and subcategories can be found in Table 2.

**Table 2:** An overview of the findings depicting categories, aspects, subcategories and domains in relation to the three documentation sources.

Category*	1.Initiating the partnership through the adolescents’ narrative	2. Establishing the partnership through adolescent-oriented communication	3. Concluding and bringing forward the partnership through goal setting
**Data sources**	Adolescents’ personal story (written)	TCs’ documentation of the person-centred dialogue	TCs’ documentation of goal setting
**Aspects**⁎⁎	Resources and capacity Risks and obstacles Need of support	Resources and capacity Risks and obstacles Need of support	Goals commonly agreed upon How to accomplish the goals
	Subcategories (Table 3) derived inductively	**HEADDS**⁎⁎⁎**domains** - Home - Education - Activity - Depression (mental health) - Drugs (substance use) - Sexuality	**Subcategories**⁎⁎⁎⁎ - Advancing knowledge and understanding - Enhancing personal control - Developing shared decision-making skills - Cultivating personal identity - Sharing knowledge and experiences to empower others

⁎ The main categories reflect the three cornerstones of the GPCC model for person-centred care (Ekman et al., 2011) used deductively as an interpretative lens through which to read and make sense of the data in all data sources.

⁎⁎ Aspects of relevance for person-centred care (resources and capacity, needs, goal setting) and adolescent-related aspects (risks and obstacles).

⁎⁎⁎ HEADDS psychosocial interview guide (Cohen et al., 1991).

⁎⁎⁎⁎ The subcategories derived deductively based on five dimensions of empowerment (Small et al. 2013).

### 3.1 Initiating the partnership through the adolescent's narrative

Partnership with the adolescents was initiated through the personal story they wrote before the first consultation. This procedure was repeated before the last consultation, which also involved a joint visit with the adult outpatient team. The adolescents’ narrative was sorted into key aspects of a person-centred and adolescent-oriented care, including resources, risks/obstacles, and needs, summarized in Table 3 with illustrating quotes.

**Table 3:** The adolescents’ narratives in relation to person-centred and adolescent oriented aspects.

Resources	Risks and Obstacles	Needs
**A belief in the future and your abilities** This included having multiple choices and possibilities for yourself. Trusting your ability to manage daily life, as well as future challenges was also raised, as well as self-esteem and a positive view of yourself. *“I have a positive view of myself, and my heart does not affect that view negatively.” (Participant 1)* **A functioning everyday life and a strong social network** In the narratives, attending school and free time were described with a focus on friends and activities like music or sports, but also extra jobs and feeling at home in your environment. Many described experiencing a good level of support from their surroundings and a strong social network, with a strong emphasis on friends and family. *“School is not a problem for me, which has given me great hopes for the future.” (Participant 2)* *“They (the family) are always there if needed and always support me if things get hard.” (Participant3)* **Personal attributes, motivation, and mindset** The adolescents often described their attributes, for example, positivity, helpfulness, or kindness but also cognitive abilities, such as productivity or being solution oriented. They also described aspects of motivation and ambition as strengths. *“Always trying to be positive and help others.” (Participant 4)* *“I think that I am great because I am kind.” (Participant 5)*	**Cognitive difficulties** Memory and attention deficits were described as obstacles by several adolescents. Another obstacle mentioned was difficulty understanding instructions. **Difficulties in social context** Some of the narratives described difficulties in social contexts that were not necessarily due to CHD. This could be rooted in shyness and depression or be about difficulties in dealing with and explaining one's needs to others. "*Having difficulty describing how I want things.*" (*Participant 6*) **Stress and worries** Several narratives described stress and worries, for example, about school and a future career, but also about needing future surgery or about fear of dying due to a heart defect. There was also concern about not being able to become a parent. Sometimes the narratives described a deeper fear for the future. *"My opportunities are small, and the obstacles are high and big as mountains.*"(*Participant 7*) **Physical limitations** Experiencing physical limitations and manifestations was described as frustrating and a barrier. For some, this led to inactivity and staying at home.	**Support for improving knowledge and skills** Several adolescents described a need for more knowledge. This included how to make a doctor's appointment or renew a prescription but could also be about knowing when to seek help in case of symptoms. "*I have no idea who to contact when I need to make an appointment and when it's serious enough to seek help."(Participant 8*) **Being accepted for who you are** Several young people described a need for acceptance of their limited physical capacity and being able to do things at their own pace, but also acceptance of cognitive difficulties or a greater risk of infections. *“I need others to understand that I have to solve things/tasks in my way and that I often forget what others have said.”* (*Participant 9*) **Need for support in everyday life** Many adolescents described various forms of support needed from their environment, such as psychosocial support and assistance in everyday life including school. One young person described a need for financial support to participate in sports activities. It also emerged that the adolescents were satisfied with the support provided by their family and friends. "*Sometimes when I´*m *down and not feeling well, I can have a hard time raising myself up again, then I´*m *grateful for my parents supporting and helping me*." (*Participant 10)*

### 3.2 Establishing the partnership through person-centred communication

This category describes the content of the psychosocial interviews carried out with HEADDS during the consultations, and relates to the aspects; resources and capacities, risks and obstacles and need of support (the content follows the HEADDS domains in **bold** down below).

#### 3.2.1 Resources and capacity

In the domain of the **home** environment, detailed information was gathered regarding the adolescents’ living conditions and family dynamics, including their relationships with family members, siblings, and other individuals. Parental involvement was high, as most adolescents lived with their parents.

Although the participants did not explicitly articulate their resources and capacities, the prevailing sentiment was that their parents and family played a pivotal role in providing substantial support in their day-to-day lives.

Within the **educational** domain, various resources influencing the adolescents’ lives beyond the healthcare context were identified, encompassing factors such as motivation, enjoyment in school, and aspirations for future goals and milestones. The adolescents predominantly expressed this by talking about their dreams and hopes for their educational and vocational trajectories, encompassing ambitions such as pursuing university studies to attain their ideal profession. While some adolescents described school as occasionally stressful and monotonous, most felt it was ultimately rewarding and believed they were on a positive path. One subset of adolescents demonstrated awareness of impending surgeries but remained steadfast in their determination to complete upper secondary school according to schedule, viewing it as a crucial step toward achieving their life goals. For some, pursuing education and achieving milestones ultimately represented a path towards independence and self-sufficiency.

In terms of **activities**, the adolescents described numerous resources and facilitators, such as friends, family, partners, and social engagements. Nearly all the participants reported engaging in physical activities (e.g. weightlifting, cardio, swimming and ball sports), often in the company of friends and family. A significant proportion mentioned participating in physically demanding sports like hockey, football, CrossFit, and high-intensity training. Some even dreamt of becoming elite athletes, but realized this might not be feasible due to their CHD. For others, their motivation for engaging in physical activities was to prevent future cardiac events and complications. Despite having different levels of fitness to their peers, most of the adolescents felt capable and believed their fitness levels did not differ much to that of their friends. The TC also sought to identify narratives highlighting low physical activity behaviours and provided tailored suggestions for these adolescents. In contrast, tailoring suggestions for adolescents engaging in high-intensity training was challenging, despite their presenting with symptoms of cardiac distress. The documentation did not elucidate the TC's recommendations in these instances.

In the realm of **mental health and depression** the adolescents did not explicitly describe resources or facilitators but rather the absence of evident symptoms. The main resource identified was a robust social network comprising friends, peers and classmates, with most adolescents citing friends and peers as their primary sources of mental health support. Regarding body image, some adolescents with surgical scars expressed indifference towards them. For those experiencing mental health and neuropsychiatric issues, beneficial factors included access to a reliable counsellor or psychologist and effective pharmacological treatments. Some acknowledged life's ups and downs but accepted these fluctuations were normal, and that they had to adapt to the challenges they faced in daily life.

Regarding **substance use**, the participants demonstrated awareness of the impact of alcohol, smoking and drugs on health and well-being. Many expressed a lack of interest in these substances, citing their active lifestyles and ongoing physical development as reasons for refraining from experimentation in this regard. While these substances were present in some social networks, others reported their absence.

In the context of **sexuality**, the sole specific resource identified was adolescents possessing knowledge and information about crucial aspects of contraception and family planning, particularly pertinent to their CHD.

#### 3.2.2 Risks and obstacles

Few risks and obstacles were identified in the **home** environment, primarily existing for adolescents in households grappling with social issues. However, concerning **education**, some participants reported challenges in concentration, specifically in reading, writing, and comprehending complex information. One subset of adolescents also described experiencing heightened self-imposed demands, posing a potential risk of **mental health** issues.

In the broader context, few obstacles were associated with **activities** and physical pursuits, as most of the participating adolescents were active. Nonetheless, some adolescents faced risks due to physical inactivity, which they attributed to time constraints, lack of motivation, and a lower level of fitness.

Concerning risk behaviours related to **substance use**, a few adolescents described engaging in social smoking, using oral tobacco, and having acquaintances involved with cannabis. Discussions also touched on the use of alcohol and the physical effects of energy drinks, as well as the use of protein supplements in the context of training, and the impact of these on the adolescents’ bodies and underlying medical condition.

#### 3.2.3 Need of support

To facilitate and enhance health-related learning, the TCs identified specific areas in which the participants required support, aligning with the domains of HEADDS. One recurrent issue observed was a lack of motivation for **physical activity**. In response, the TCs initiated conversations addressing ways of supporting physical activity, offering information on health-promoting behaviours (such as daily walks) and emphasizing the importance of physical activity. They encouraged the adolescents to explore activities they enjoyed and that were tailored to their abilities. However, barriers to physical activity, such as time constraints, lower levels of fitness and less energy than their peers, were frequently raised as issues. For some adolescents, the TCs played a coordinating role, facilitating team collaboration and offering referrals to physiotherapists when requested. Specific TC plans outlined the need for information regarding strenuous physical activities like heavy weightlifting, running, sports, and exercise, exploring their impact on CHD. Questions about vertigo and expressions of illness symptoms related to intense physical activity were also addressed.

Regarding **substance use**, the TCs provided comprehensive information on smoking, vaping and oral tobacco. The adolescents asked about alcohol, and specifically about its effects in relation to CHD and potential risks. Conversations between the TCs and adolescents revealed the prevalence of illegal drug usage in their social circles and schools, although the extent of individual involvement remained unclear.

The adolescents commonly expressed **mental health** concerns, including worry/anxiety, stress and depression and linked these to stress in school, general life pressures, relationships with peers, high expectations of self, and, for one subgroup, neuropsychiatric conditions. Worries extended to various aspects of their CHD, such as overall health, impending surgeries, the transition to adult care and lifespan. In their meetings with the adolescents, the TCs focused on understanding these concerns and providing suggestions for coping strategies.

Within the domain of **sexuality**, discussions between TCs and adolescents were focused on CHD-related considerations, including the use of contraceptives and their impact on CHD, implications of pharmacological treatment, hereditary aspects of CHD, family planning and pregnancy. The TCs referred the adolescents to youth reception services for contraceptives or discussions on related matters as needed.

### 3.3 Concluding and bringing the partnership forward through goal setting

Goal setting was a concluding and essential part of the consultations. The jointly documented transition plans outlined a spectrum of resources and capabilities, encompassing personal traits like curiosity, sociability, and effective communication skills. A solid foundation of self-awareness regarding personal capacities and learning needs contributed to the adolescents’ development of knowledge, understanding, and independence. The documentation and structure of the goalsetting process is summarized below:

#### 3.3.1 Goals commonly agreed upon

##### 3.3.1.1 Advancing knowledge and understanding

One goal was to increase the adolescents’ knowledge and understanding of their CHD. This involved deepening comprehension of their medical history with knowledge about previous interventions and treatments and helping them consider future expectations and consequences for overall health. There was a spectrum of curiosity, with some adolescents seeking overarching facts about their CHD, while others wanted a more comprehensive grasp of the physiology and potential complications. Having a deep understanding of their condition would help them formulate relevant questions during medical visits and could contribute to their more effectively communicating information to others.

##### 3.3.1.2 Enhancing personal control

The goal of improving personal control encompassed a range of aspects. Supporting personal autonomy involved practising attentiveness and maintaining focus both prior and during medical appointments. Taking on increased responsibility and active participation included managing practicalities, like scheduling appointments, arranging transportation and attending medical appointments. Proficiency in navigating adult healthcare systems was built by discussing where to turn in various situations, while strengthening self-management involved actively overseeing prescriptions, managing daily medication routines, and autonomously formulating questions before and during appointments.

According to the formulated goals, it was essential for the adolescents to develop the ability to prioritize critical aspects related to their CHD and also acknowledge personal limitations. In order to effectively cope with their concerns about future medical interventions, they had to communicate efficiently with others, as well as plan strategies to manage such concerns. Establishing sustainable health strategies entailed maintaining physical activity and adopting health-conscious behaviours crucial for adult life, such as those relevant to pregnancy and future health considerations.

##### 3.3.1.3 Developing shared decision-making skills

A set of goals was established to enhance and develop shared decision-making skills. For the adolescents, greater independence meant taking control of scheduling appointments and attending visits without parents. Additionally, their efforts to fortify relationships with healthcare providers could be realized by actively engaging during appointments, posing questions and generally communicating proactively. It was evident that their independence and active participation during medical visits had evolved between the ages of 16–18. During this period, noticeable progression was observed, indicating a substantial improvement, and honing of these essential skills. Nevertheless, the transition plans reflected varying patterns, from a progression towards increased involvement over time to less apparent progress.

##### 3.3.1.4 Cultivating personal identity

Several goals circled around nurturing awareness and confidence in the adolescents’ sense of self, particularly in the context of a gradual transition into adulthood. Promoting health and quality of life was pivotal, underscored by exploring suitable and engaging physical activities that would impart a sense of belonging with their peers. This not only contributed to the adolescents’ overall well-being but also played a crucial role in fostering self-assurance and heightened consciousness of personal identity.

##### 3.3.1.5 Sharing knowledge and experiences to empower others

Goals addressing sharing of knowledge and experiences to help peers in similar situations were identified. It was crucial the adolescents possessed sufficient and pertinent knowledge about their CHD to facilitate effective communication in diverse settings, ranging from interacting with primary care providers to engaging with teachers and their social network. Additionally, some adolescents expressed a need for emotional support, valuing the reassurance that comes from sharing thoughts and having a reliable presence when they need it.

#### 3.3.2 How to accomplish the goals

This subcategory describes the measures the adolescents needed to take in order to achieve the goals they had agreed upon. This encompassed acquiring knowledge about their specific type of CHD, including its name, treatment options, interventions, and potential consequences. Such foundational knowledge was essential for the adolescents to comprehend the CHD's impact on daily life and effectively communicate this information to others.

The adolescents often sought help with reminders related to self-monitoring, encouragement in maintaining physical activity, and keeping track of healthcare visits. To prepare for healthcare visits, they engaged in self-directed learning by exploring provided materials, such as written information, websites, and visuals detailing their CHD (pictures). Prior to these visits, they took proactive steps and utilized their mobile devices as organizational tools, noting down questions for healthcare providers and storing important health-related information. During the visits, the adolescents made collaborative efforts to summarize and discuss with the HCP the information they had acquired. Their mobile devices served as versatile tools, not only for organizing questions but also as digital notebooks for health-related activities, medication reminders, prescription details, scheduling visits and storing contact information for healthcare providers.

Some adolescents were keen on parental support as a resource, using parents as valuable collaborators in exchanging thoughts, planning healthcare visits, and navigating various aspects of their health journey. Motivation emerged as a critical resource, frequently documented as a driving force for achieving goals related to increased physical activity and adopting healthier life habits.

## 4 Discussion

Problematizing the evaluation of PCC in a program originally based on the same concept, such as the Stepstones transition program, involves questioning the necessity and effectiveness of such evaluations. It could be argued that the program inherently embodies person-centredness, making evaluation redundant. PCC and transition programs are complex interventions, necessitating evaluation of how crucial aspects of the intervention's program theory are implemented within the trial, thus informing subsequent improvements to similar interventions (Skivington et al., 2021). The provision of PCC is a growing imperative across healthcare settings (Britten et al., 2017) and entails a shift in the focus of care, from a traditional medical perspective to a more health-oriented approach. More specifically, it requires identification of the person's goals rather than the health care system's goals, emphasizing the person's own resources and their formulated need for support (Jansson et al., 2018) and to what extent these assumptions are reflected in the health care documentation. Below we critically assess the PCC practices provided and documented in the Stepstones transition program to ensure meaningful and effective implementation of PCC in the future, as well as facilitate an understanding of the challenges of doing so for adolescents with CHD.

### 4.1 How did the transition program capture the adolescents’ narrative?

Prior to the first visit with the TC, the adolescents’ written personal narratives captured their own view of their resources, obstacles and need of support in relation to their condition and its impact on everyday life. The narratives served as an important tool in their conversations during these visits. In contrast, the transition plans, written by the TCs, served as documentation for the care arrangements in consultations with the participants. Use of the HEADDS psychosocial interview guide helped steer the conversation to topics relevant to the adolescents’ developmental stages, while also capturing a holistic view of their lives. In the transition plans, the way the HEADDS documentation was formulated made it challenging to discern who had initiated the conversation topics and set the agenda, meaning the narrative and person-centredness of many elicitations could not be adequately assessed. However, the topics emerging in the adolescents’ written narratives did reflect the goals agreed upon, indicating that these narratives constituted the basis for the co-creation of the transition plan. Such findings are confirmed in similar studies, highlighting the challenges of creating person-centred documentation (Jansson et al., 2018; Lydahl et al., 2022). In our study, we were able to triangulate data sources, allowing comparison between sources and potentially compensating for the inherent limitation of each data source (Bekhet and Zauszniewski, 2012). Moreover, the process evaluation of the Stepstones transition program concluded that the consultations between the adolescents and TC were (in the adolescents’ experience) characterized by being seen as a unique person in a safe space (Bekhet and Zauszniewski, 2012) and that the PCC components were delivered with high fidelity (Saarijärvi et al., 2022). Thus, the notion that the program had been interpreted as person-centred from a variety of perspectives was reinforced in its implementation.

### 4.2 How was the partnership supported by the transition program?

From the documentary analysis it was clear that setting personal goals for the adolescents and formulating goals for their transfer and transition largely focused on different domains of patient empowerment that were all relevant to their condition, i.e. healthcare transition and everyday life. In addition, the use of HEADDS gave the consultations in-depth access to the adolescents’ personal story, how they viewed themselves and their health, and the associated challenges and risks. However, due to the nature of the data, the process of formulating goals and who was driving this process are not clear. Previous studies show that the adolescents viewed goal setting within the Stepstones transition program as a collaborative process in which the TC guided the adolescent in finding goals that were relevant and feasible to them and their context (Saarijärvi et al., 2021). Thus, this approach is in line with PCC being two experts meeting in a shared decision-making process (Ekman et al., 2011). Our findings also show that using HEADDS can be one method to promote person-centredness for this population, as it emphasizes communication and is robustly founded within adolescent health and medicine (Christie and Viner, 2005). Recent findings from a systematic review indicate that adolescents prefer a self-administered digitally delivered tool based on HEADDS rather than a face-to-face psychosocial interview that requires personal disclosure (Waller et al., 2023). However, although this might help capture the adolescent's narrative, it is probably less effective for creating a partnership.

Besides psychosocial assessment it is important to help the adolescents gain a deep understanding of their condition, as this helps them formulate relevant questions during medical visits and could contribute to their more effectively communicating information to others. Such an understanding is a prerequisite for building a partnership because it empowers the adolescents to actively participate in their healthcare journey. In the evaluation of the Stepstones transition program, its primary and secondary outcomes in terms of improved level of empowerment and disease-related knowledge supported this assumption (Bratt et al., 2023). Our research group has also shown that a higher level of transition readiness predicts a higher level of patient empowerment (Acuña Mora et al., 2022).

**Fig. 2:**
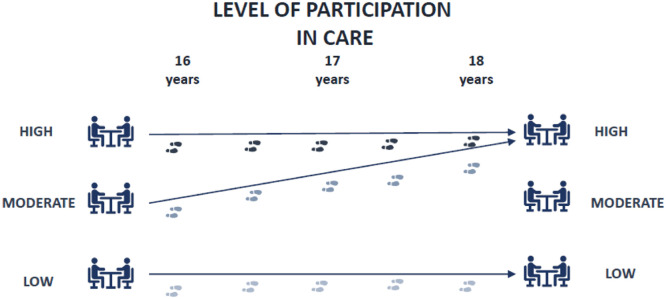
Indication of trends regarding participants' involvement in care.

The documentation of adolescent participation in care revealed diverse patterns in relation to independence and involvement in the care process. Most transition plans indicated a progression toward increased involvement over time, emphasising clear recognition of the importance of evolving towards greater autonomy in aspects such as self-management, parental engagement, and interactions with healthcare providers. Conversely, some transition plans depicted less apparent progress. This discrepancy can be attributed to two distinct scenarios: the first involves a high initial level of participation that remains relatively constant over time, while the second scenario portrays individuals starting with a lower level of involvement, with less obvious improvement throughout the documented period. For visual representations of these various trajectories of adolescent participation in care, see Fig. 2.

### 4.3 How does the documentation in the transition program support the implementation of a person-centred approach?

According to the GPCCs model of PCC, documentation is the way to safeguard the partnership (Ekman et al., 2011). The three data sources supported the implementation of a PCC approach in various ways. Both the adolescents’ story and their assessment of needs, risks and resources (through the psychosocial interview) assisted in identifying their individual narratives and accessing their wishes and resources. Öhlen et al. (Öhlén et al., 2022) state that “it is through narrative‐in‐action that wishes and resources are created, shaped and negotiated” (p.2). However, this is captured by the documentation in the transition program to varying extents, as discussed below.

Documented examples from the transition plans highlighted the necessity of support from healthcare professionals, encompassing the provision of relevant materials illustrating the CHD, ensuring the availability of channels for contacting HCPs between scheduled visits, and fostering an environment in which adolescents are actively encouraged to pose questions during their appointments. One example of the documentation not being clear about the use of a person-centred approach arose with adolescents engaging in high-intensity training despite symptoms of cardiac distress. In these instances, the documentation did not elucidate the TC's recommendations, leaving a gap in understanding the approach taken. It is possible that these situations required medical consultation, which was therefore documented in the medical record. Within the framework of the RCT, transition plans were not part of the regular medical records. Lydahl et al. (Lydahl et al., 2022) advocate for prioritizing life-world goals to enable alignment with medical/health goals, making both perspectives essential and relevant for patients. This should consequently be considered in real-world clinical practice.

Motivation to achieve goals related to increased physical activity and the adoption of healthier life habits could be further boosted by the use of apps that serve as reminders and monitoring tools, offering valuable support in tracking health status and behaviours. This comprehensive approach ensures that transition plans are tailored to strengths and needs, fostering a more effective and person-centred transition process.

As indicated in the transition plans, one subset of adolescents required minimal support to achieve their goals, while others actively sought support to reach greater independence. However, it is noteworthy that a few faced the challenge of overprotective parents, hindering their desire for increased autonomy. In line with previous studies (Coyne et al., 2019; Heath et al., 2017), the documentation underscores the diverse support needs among adolescents in the transition process, emphasizing the importance of tailoring support structures to individual preferences and circumstances.

### 4.4 Limitations

This paper is based on documentation collected within an RCT, written by TCs trained in PCC. However, only the adolescents’ written narratives were based on first-hand information. This has implications for the depth of analysis and the ability to explore emerging phenomena. Moreover, the selection of documents for analysis may introduce bias if certain types of documents are favoured over others. In this paper, we included a diverse range of sources to minimize bias in the analysis and analysed the full data set for all three sources.

A few risks and obstacles were identified in the home environment, primarily for adolescents residing in households grappling with social issues. However, concerning education, some participants reported challenges in concentration, specifically in reading, writing, and comprehending complex information. Previous studies evaluating research participation in adolescents with chronic conditions have shown that adolescents from socio-economically disadvantaged backgrounds tend not to participate in research to the same extent as those from affluent households (Roick et al., 2018). The sample representing the Stepstones RCT had the same issue (Bratt et al., 2023; Saarijärvi et al., 2020). Our results must therefore take this into consideration, as the concept of person-centredness is culturally and socio-economically dependent (McWilliam, 2009; Pulvirenti et al., 2014).

Finally, evaluating PCC within its original program framework may introduce assessment bias, as the stakeholders involved in the program's development may be inclined to perceive it more positively, potentially skewing evaluation results. However, involving authors with various roles in the evaluation of the program contributed with different perspectives.

### 4.5 Conclusions

The evaluation of the documentation of this transition program indicates that it was successful in capturing the adolescents’ narratives through their written stories and the HEADDS psychosocial interview, which informed goal setting and transition planning. However, the documentation made it difficult to discern who initiated topics and set the agenda, challenging the assessment of person-centredness. The program supported partnership through collaborative goal setting focused on patient empowerment across different life domains. Using HEADDS facilitated understanding of the adolescents’ perspectives and promoted shared decision-making, aligning with PCC principles. These findings might be helpful when implementing transition programs in clinical practice as improved empowerment and disease knowledge outcomes suggest the program effectively built partnership. Overall, the program implemented several PCC components, such as eliciting narratives, collaborative goal setting, and tailoring support needs. However, the documentation had limitations in fully reflecting the person-centred practices employed, highlighting opportunities for improvement in person-centred documentation.

## Funding sources

The work presented in this article was supported by research grants from (a) the Swedish Heart-Lung Foundation (grant 20150535); (b) Swedish Research Council for Health, Working Life and Welfare-FORTE (grant STYA-2015/0003); (c) the Swedish Children's Heart Association; (d) the Swedish Research Council (grant 2015e02503); (e) the Institute of Health and Care Sciences, Sahlgrenska Academy, University of Gothenburg in Sweden; (f) Department of Neurobiology, Care Sciences and Society, Karolinska Institute, Stockholm, Sweden. The funding bodies have not participated in the study design, collection and analysis of data or writing the manuscript.

## Data availability

The data that has been used is confidential. Due to the sensitive nature of the documentation from the consultations used in this study, the data would remain confidential and not be shared.

## CRediT authorship contribution statement

**Åsa Burström:** Writing – review & editing, Writing – original draft, Visualization, Validation, Methodology, Investigation, Formal analysis, Data curation, Conceptualization. **Markus Saarijärvi:** Writing – review & editing, Writing – original draft, Visualization, Validation, Methodology, Investigation, Formal analysis, Data curation, Conceptualization. **Sandra Skogby:** Writing – review & editing, Validation, Methodology, Formal analysis, Data curation. **Anna Lena Brorsson:** Writing – original draft, Validation, Methodology, Formal analysis, Data curation. **Ewa-Lena Bratt:** Writing – review & editing, Writing – original draft, Validation, Methodology, Investigation, Funding acquisition, Formal analysis, Data curation, Conceptualization. **Carina Sparud-Lundin:** Writing – review & editing, Writing – original draft, Visualization, Validation, Methodology, Investigation, Funding acquisition, Formal analysis, Data curation, Conceptualization.

## Declaration of competing interest

The authors have no conflict of interest.

## Appendix Supplementary materials

Image, application 1
